# Digital health in palliative care: use is largely limited to conventional technologies – a cross-sectional survey of healthcare professionals

**DOI:** 10.1186/s12913-026-14990-5

**Published:** 2026-06-23

**Authors:** Felix Muehlensiepen, Robert Darkow, Larissa Fink, Yuriy Ignatyev, Anne Gehlhaar, Anke Lauke, Marcel-Alexander Kamp, Martin Heinze, Matthew Allsop, Susann May

**Affiliations:** 1 Center for Health Services Research, Faculty of Health Sciences Brandenburg, Brandenburg Medical School, Seebad 82/83, 15562 Rüdersdorf, Brandenburg Germany; 2 Deutsches Herzzentrum der Charité – Department of Cardiology, Angiology and Intensive Care Medicine, Charitéplatz 1, 10117 Berlin, Germany; 3 Charité – Universitätsmedizin Berlin, Corporate Member of Freie Universität Berlin and Humboldt-Universität zu Berlin, Charitéplatz 1, 10117 Berlin, Germany; 4 University Grenoble Alpes, CNRS, Grenoble INP, LIG, SANGRIA, Grenoble, 38000 France; 5 FH Joanneum - University of Applied Sciences, Health Studies, Graz, Austria; 6 Department of Oncology and Palliative Medicine, Brandenburg Medical School, Immanuel Klinik Rüdersdorf, 15562 Rüdersdorf, Brandenburg Germany; 7 Department of Psychiatry and Psychotherapy, Brandenburg Medical School, Immanuel Klinik Rüdersdorf, 15562 Rüdersdorf, Germany; 8 Academic Unit of Palliative Care, Leeds Institute of Health Sciences, University of Leeds, Leeds, LS2 9JT UK

**Keywords:** Palliative care, Digital health, Survey, Health personnel, Technology adoption, Health literacy, Health services research

## Abstract

**Background:**

Digital health technologies (DHTs) can help address challenges in palliative care, particularly in rural and underserved areas. However, evidence on their routine use and acceptance among healthcare professionals (HCPs) remains limited.

**Aim:**

To assess HCPs reported use of DHTs, digital health literacy, attitudes, and perceived benefits and barriers regarding DHT implementation in routine palliative care.

**Design:**

Cross-sectional survey using a self-administered questionnaire informed by prior qualitative research. Data were collected online and on paper (Sept–Nov 2023).

**Setting/participants:**

152 HCPs in palliative care across Brandenburg, Germany: 68 physicians, 36 nurses, and 48 others (e.g. psycho-oncologists, social workers).

**Results:**

Physicians showed the highest digital health literacy (mean eHEALS: 33/40). Nurses expressed the highest general agreement with DHTs, though acceptance in palliative care was lower—especially among physicians. Email (72–78%) and documentation tools (60–67%) were widely used. Innovative tools such as video consultations (6%), mobile apps (10–22%), and wearables (10–22%) were used infrequently. Perceived benefits included location flexibility (up to 81%) and time independence (up to 78%). Barriers included inadequate infrastructure (up to 78%) and limited digital skills among patients and families (up to 80%). DHT use was more frequent in inpatient settings (ρ = 0.183, *p* = 0.014), urban areas (*p* = 0.04), and among physicians and nurses (*p* = 0.049). Non-users had a higher median age than users (*p* = 0.023). Those using conventional tools were more likely to adopt innovative ones (*p* < 0.001).

**Conclusions:**

DHT use in palliative care remains centered on conventional tools. While professionals recognize potential benefits, structural deficits and limited perceived relevance hinder adoption. Strengthening infrastructure and digital literacy is key to sustainable digital transformation.

**Supplementary Information:**

The online version contains supplementary material available at 10.1186/s12913-026-14990-5.

## Key statements

What is already known about the topic?

What this paper adds

Implications for practice, theory or policy

- Digital health technologies (DHTs) can support interdisciplinary care and improve access to information and symptom monitoring in palliative care.
- The principle of “low tech — high touch” in palliative care may limit the perceived relevance of DHTs.

- DHT use in palliative care is largely limited to conventional technologies, while innovative tools (e.g. video consultations, mobile apps) are rarely adopted.
- DHT uptake is significantly higher in inpatient and urban settings and is associated with younger age among healthcare professionals.
- Professionals who use conventional technologies are more likely to adopt innovative tools, while general attitudes do not predict actual usage.

- Digital transformation strategies should build on existing digital routines to support the uptake of more advanced technologies.
- Investments in infrastructure and tailored digital literacy support are essential to close gaps in access and competence.
- Implementation efforts must account for setting-specific and demographic factors to ensure equitable and context-sensitive integration of DHTs.

## Background

As populations age, demand for palliative care is increasing across health systems [1]. In Germany, this trend is mirrored by regional disparities, particularly in rural areas such as Brandenburg, which face limited access to specialized palliative care, long travel distances, and a growing shortage of healthcare professionals (HCPs), including nurses and palliative care specialists [2–4]. The uneven involvement of general practitioners in primary palliative care further contributes to these gaps [5–8].

Digital health technologies (DHTs) can support interdisciplinary care and improve access to information and symptom monitoring in palliative care [9]. DHT-supported interventions include information provision, symptom tracking, psychological support, and communication tools. These have shown positive effects on pain management, symptom distress, quality of life, and patient satisfaction [9]. Amid workforce shortages and rising care demands, DHTs may help optimize resources and ease burdens on professionals and caregivers [10, 11]. Evidence also links targeted digital implementation to better care quality and service coordination [12].

In a meta-review, Finucane et al. [13] found that common DHTs in trials in the palliative care domain include videoconferencing (17%), electronic health records (16%), and phone calls (13%). Still, the field often follows a “low tech – high touch” ethos, prioritizing human connection over technological solutions. This principle resonates with patients, families, and providers [10, 11].

Despite growing interest, data on the actual use and acceptance of DHTs in German palliative care remain scarce. Acceptance, usability, and ethical alignment must be considered in this sensitive context.

This study addresses this gap through a survey, asking:

1. Which digital technologies are currently used in hospice and palliative care?
2. How are they accepted by HCPs?
3. What additional applications are considered useful?
4. What is the level of digital health literacy?
5. What barriers are perceived regarding DHT use in palliative care?”

## Methods

This cross-sectional survey collected self-completed online or paper-based data from palliative care HCPs in Brandenburg, Germany.

### Study setting

Brandenburg, a sparsely populated federal state with 2.5 million inhabitants, has a high proportion of older residents and limited access to palliative care [5, 14].

### Participants and data collection

The survey targeted physicians, nurses, and other palliative care professionals (e.g., psycho-oncologists, therapists, coordinators). Inclusion criteria: (1) HCP in palliative care, (2) based in Brandenburg, (3) active (not retired/in training).

The survey investigated the sociodemographic characteristics of HCPs, their digital health literacy, the use of digital technologies in daily practice, as well as attitudes toward DHTs and perceived benefits and barriers in palliative care. The questionnaire draws on findings from two preliminary qualitative studies involving healthcare professionals, patients, and relatives in palliative care contexts [10, 11]. It comprises the following sections:

- Sociodemographic data: This section captured gender, age, profession, years of experience, care setting, and municipality size.
- Digital health literacy: Measured using the 8-item eHEALS scale [15], this section assesses perceived skills in locating, evaluating, and applying online health information, rated on a 5-point Likert scale.
- Using digital technologies in daily practice: Participants reported which digital tools (e.g., emails, video consultations, documentation software, apps, wearables) they currently use and for what purposes.
- Attitudes towards digital technologies: This section explored participants’ general and palliative care–specific attitudes, including perceived appropriateness and changes in views due to the COVID-19 pandemic.
- Potentials and barriers of digital technologies: Participants were asked to indicate potential benefits (e.g., flexibility, time savings) and barriers (e.g., infrastructure, data security, user-friendliness) related to DHTs. A detailed description of the item definitions used in the questionnaire can be found in the appendix (Supplementary Material 1).

The questionnaire was piloted with five researchers and five practitioners and revised until it was clear to all. The final questionnaire could be completed using LimeSurvey, a web-based survey tool, or in a paper pencil-based form. The survey was conducted over a 3-month period between September and November 2023 in accordance with the checklist for reporting of survey studies (CROSS) [16]. Supplementary Material 2 contains the final version of the questionnaire.

We distributed the survey across various healthcare settings involved in palliative and hospice care in Brandenburg, including specialised outpatient palliative care teams (SOPC), inpatient and outpatient hospices, palliative care units, cancer counselling centers, and outpatient physicians. Institutions received multiple copies of the questionnaire (physicians received one each), and we also offered an option for online participation. Three weeks later, we sent reminder postcards to all previously contacted institutions. Additionally, we promoted the survey at an event hosted by the Landesarbeitsgemeinschaft Onkologische Versorgung Brandenburg (LAGO). The LAGO is a non-profit association that brings together oncological institutions, professional groups, self-help organizations, and volunteers to improve oncological care in the federal state of Brandenburg. Interested individuals participated on site, and LAGO returned the completed questionnaires for evaluation. Participants provided written consent after receiving study information. The study team collected all completed questionnaires.

### Statistical Analysis

Statistical analyses were performed using SPSS Statistics for Windows, version 29 (IBM Corp), with significance levels set at *p* < 0.05 [17]. One-tailed tests were applied only where directional hypotheses had been specified a priori on the basis of prior qualitative findings [10, 11], namely that physicians and nurses would use DHTs more frequently than other professional groups and that non-users would tend to be older; all other comparisons were two-tailed. Descriptive statistics included frequencies and percentages for categorical variables, median scores and ranges for ordinal single-item variables, and means for summed eHEALS scores.

The analytical strategy was structured to address each research question: RQ1 (current DHT use): frequencies and percentages; RQ2 (acceptance and attitudes): descriptive distributions of attitude items and Mann-Whitney U comparisons between user groups; RQ3 (useful applications): multiple-response frequencies of perceived potentials; RQ4 (digital health literacy): summed eHEALS scores reported by professional group; RQ5 (perceived barriers): multiple-response frequencies of barrier items.

Data preparation included coding data categories in the following way: Care structure was categorized into five settings: (1) outpatient care, (2) outpatient hospice care, (3) inpatient hospice care, (4) inpatient care, and (5) other. Urbanization level was measured on a five-point scale, ranging from rural areas (1) to metropolitan cities (5). Utilization of care resources referred to the number of different DHTs and services participants reported using in their professional practice (e.g., email, video consultations, documentation tools). This was operationalized by summing the total number of technologies used. General attitude toward digital technologies was measured using a Likert scale, assessing participants’ agreement with statements on the usefulness and relevance of DHTs in palliative care. Professional background included profession (coded: physician = 3, nurse = 2, other healthcare professionals = 1, volunteers = 0), years of experience, employment status, and digitalization affinity (coded). Age was recorded in completed years and analysed as a continuous variable. An additional item on practice setting (single-handed practice, group practice, medical care centre, or hospital) was recorded but not used in the analyses, as it was applicable mainly to physicians in ambulatory care and therefore had high item-level missingness; care structure was retained as the structural variable.

Prior to factor analysis, sample adequacy was assessed using the Kaiser-Meyer-Olkin (KMO) measure and Bartlett’s test of sphericity. A principal component analysis (PCA) with varimax rotation was conducted to identify latent factors among demographic and professional variables. Rather than being entered as factor scores in regression models, the three extracted components were used to guide the selection and grouping of individual variables for the subsequent bivariate analyses (Spearman’s rank-order correlation, Mann-Whitney U tests, and ANOVA). To examine associations between care structures, urbanization levels, and utilization patterns, Spearman’s rank-order correlation was applied. Differences between users and non-users of care resources were tested using the Mann-Whitney U test (MWU). The influence of urbanization on care structure and resource utilization was further analyzed using ANOVA.

## Results

### Participant characteristics

Of 224 contacted institutions, 152 HCPs participated: 68 physicians, 36 nurses, and 48 others (e.g., psycho-oncologists, social workers, coordinators). Most had > 20 years’ experience, especially physicians (63%) and nurses (50%). Female participants predominated among nurses (83%) and other HCPs (94%). The majority were aged 51–60 and worked in towns with <100,000 inhabitants. Outpatient care was most frequently reported; hospice care was more common among other HCPs. Physicians were often self-employed (47%), while nurses (89%) and other HCPs (85%) were mainly employed. See Table 1 for details.

**Table 1:** Participants characteristics; *N* = 152; *multiple answers possible

	Characteristics	Physicians (N = 68)		Nurses (N = 36)		Health Care Professions (N = 48)	
		n	%	n	%	n	%
1	Duration of professional activity						
	<10	2	3	6	17	11	23
	10–20	20	29	8	22	8	17
	21–30	23	34	11	31	13	27
	31–40	16	24	7	19	9	19
	>40	2	3	1	3	0	0
	Missing	5		3	8	7	15
2	Gender						
	Female	39	57	30	83	45	94
	Male	21	31	2	6	3	6
	Non-binary	1	1	0	0	0	0
	Missing	7	10	4	11	0	0
3	Age						
	<40	4	6	5	14	9	19
	40–50	16	24	11	36	7	16
	51–60	31	46	16	44	21	44
	61–70	13	19	1	3	9	19
	>70	1	1	0	0	2	4
	Missing	3	4	3	8	0	0
4	Number of inhabitants in the locale in which the participant works						
	<5.000	9	13	3	8	2	4
	5–20.000	23	34	17	47	18	38
	20.001–100.000	20	29	12	33	24	50
	100.001- 1Mio	12	18	1	3	3	6
	>1 Mio	1	1	1	3	0	0
	Missing	3	4	2	6	1	2
5	Setting						
	Standalone practice	13	19	3	8	6	13
	Practice partnership	15	22	3	8	0	0
	Medical care center	6	9	1	3	0	0
	Hospital	7	10	6	17	2	4
	Missing	27	40	23	64	40	83
6	Employment relationship						
	Self-employed	32	47	2	6	2	4
	Employed	24	35	32	89	41	85
	Self-employed and employed	9	13	0	0	2	4
	Missing	3	4	2	6	3	6
7	Care Structure*	6	9	6	17	12	25
	Inpatient Palliative Care	43	63	19	53	12	25
	Outpatient Palliative Care	7	10	9	25	22	46
	Hospice (inpatient & outpatient)	9	13	0	0	5	10
	Others						

### eHEALS

Digital health literacy was generally moderate to high, highest among physicians (mean: 33), followed by nurses (31.5) and other HCPs (30.5).

### Use of digital health technologies

**Fig. 1:**
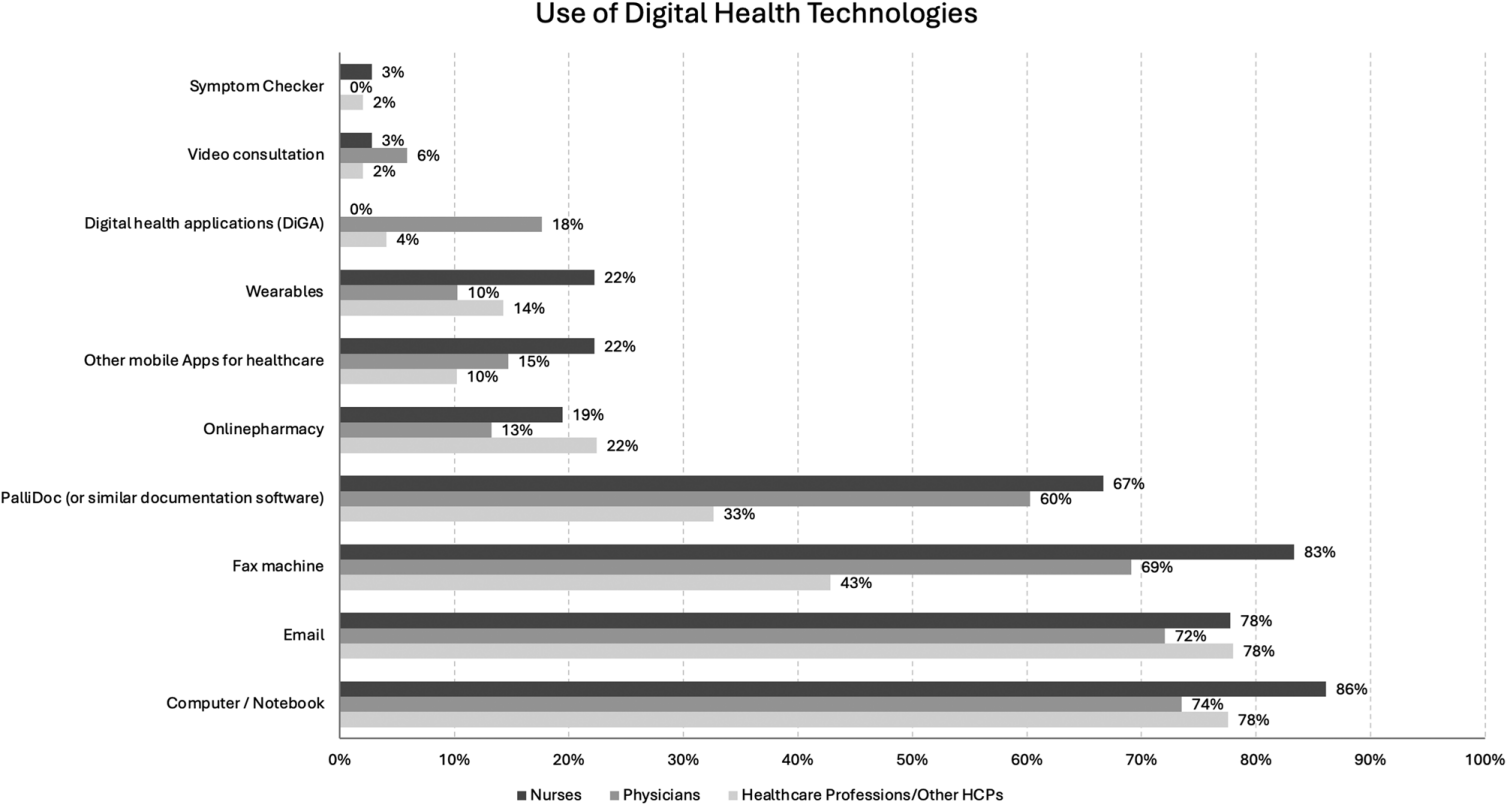
Assessment of the current use of digital health technologies; *N* = 152; data in percent

Conventional tools such as email and computers were widely used, especially among nurses. In contrast, video consultations, apps, and wearables were rarely used. Nurses reported the highest mobile app use (22%). Fax machines were still widely used, particularly among nurses (83%). Documentation tools (e.g., PalliDoc) were common among physicians and nurses. See Fig. 1.

### Appropriateness of digital health technologies

**Fig. 2:**
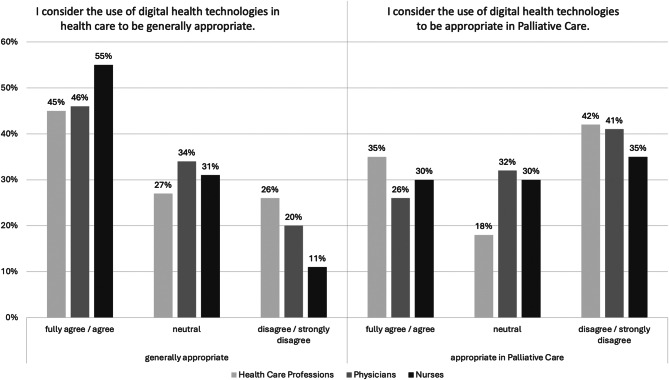
Assessment of the appropriateness of digital health technologies in general and in palliative care context; *N* = 152; data in percent

DHTs were considered generally appropriate by 45–55% across groups, but less so in palliative care, with agreement dropping to 26–35%. Physicians expressed the highest levels of disagreement. See Fig. 2.

### Changes in attitudes toward digital health technologies due to COVID-19

**Fig. 3:**
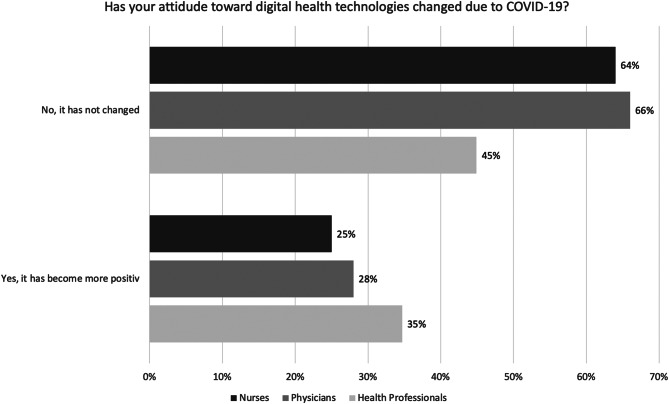
Assessment of changes in attitudes toward digital health technologies due to COVID-19; *N* = 152; data in percent

While 25–35% reported a positive shift in attitudes, most stated no change. Physicians and nurses were least affected. See Fig. 3.

### Perceived potentials of using digital health technologies

**Fig. 4:**
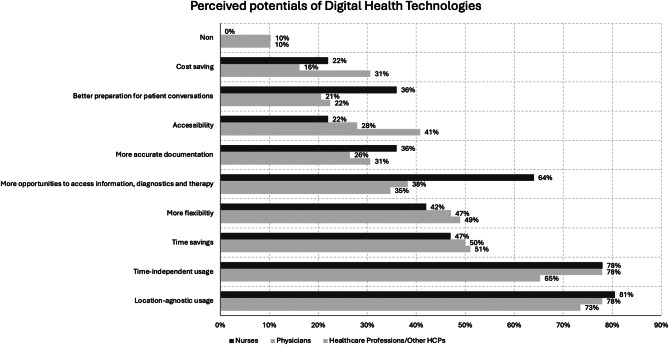
Assessment of perceived potentials of digital health technologies; *N* = 152; data in percent: multiple answers possible

Participants saw key benefits in flexibility (location and time), time savings, and access to information. Nurses most frequently reported benefits related to patient interaction and documentation accuracy. Cost savings and improved accessibility were seen as secondary. Few reported no benefit. See Fig. 4.

### Perceived drawbacks of using digital health technologies

**Fig. 5:**
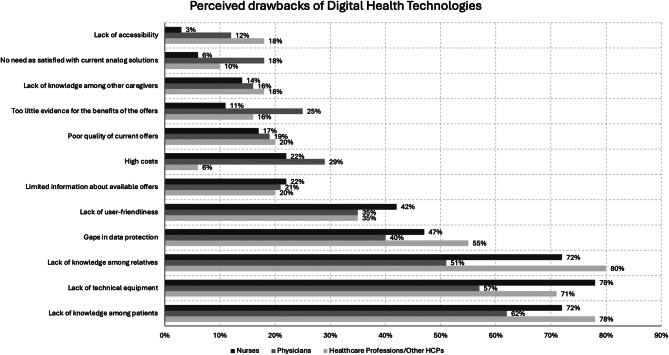
Assessment of perceived drawbacks of digital health technologies; *N* = 152; data in percent: multiple answers possible

Main barriers were insufficient infrastructure and limited digital skills among patients and families. Data protection, usability, and lack of evidence were additional concerns, especially among physicians. Cost and accessibility played a minor role. See Fig. 5.

### Patterns of using digital health technologies in palliative care

To explore associations between individual and structural factors and DHT use, a factor analysis was conducted. Sampling adequacy was confirmed (KMO = 0.691; Bartlett’s test: χ^2^ = 160.672, *p* < 0.001). Three factors emerged: Factor 1: Age and years of professional experience; Factor 2: Profession, gender, and employment status; Factor 3: Digital affinity and place of work (urban/rural). Together, the three components explained 65% of the total variance (Factor 1: 28%; Factor 2: 20%; Factor 3: 17%).

DHT use was significantly higher in inpatient settings (Spearman’s ρ = 0.183, *p* = 0.014) and urban areas. The larger the city, the more likely participants were to work in inpatient care (ρ = 0.140, *p* = 0.048) and to use a broader range of digital tools (*p* = 0.04).

Users and non-users differed significantly: Physicians and nurses were more likely to use DHTs than other professionals or volunteers (MWU = 967, *p* = 0.049, one-tailed). Non-users had a higher median age than users (56 [IQR 53–62] vs. 54 [IQR 45–58] years; Mann–Whitney *U* = 922, Z = −1.99, *p* = 0.023, one-tailed). No significant differences in general attitudes (MWU *p* = 0.13) or place of work (MWU *p* = 0.41). Conventional technologies (e.g., email, desktop computers, fax, clinical documentation tools) were used significantly more often than innovative technologies (e.g., video consultations, mobile apps, wearables) (MWU = 14,532.5, *p* < 0.001). Importantly, those using conventional tools were also significantly more likely to adopt innovative ones (ANOVA *p* < 0.001). No significant correlation was found between age and attitudes, nor between care structure and use (MWU = 1054.5, *p* = 0.13). Practice setting was not analysed as a separate variable because of its high missingness and overlap with care structure.

## Discussion

This study highlights that the use of DHTs in palliative care remains limited, with traditional tools such as email and fax still dominating, particularly among non-physician professionals and in rural settings. While healthcare professionals recognise potential benefits, such as improved flexibility and documentation, uptake is hindered by technical barriers, data protection concerns, and the digital literacy of healthcare professionals. Attitudes toward DHTs vary by role, with physicians being more skeptical and nurses more accepting. Overall, digital readiness appears to influence both usage and openness to adopting more innovative tools. Drawing on perspectives from physicians, nurses, and other healthcare professionals across diverse care settings, the findings reveal key barriers and enablers to digital transformation in palliative care. Although the survey was conducted in Germany, the results offer valuable insights for palliative care systems internationally, particularly in regions facing similar demographic, infrastructural, and workforce challenges.

### Principal findings

This study examined the use, acceptance, and perceived potential of DHTs in palliative care among HCPs in Brandenburg. While basic digital tools such as email and clinical documentation systems are widely used, innovative technologies like video consultations, mobile apps, and wearables remain rare. DHT use was significantly more common in inpatient settings, urban areas, and among physicians and nurses, while non-users were more likely to be older or in non-medical roles. Despite relatively high digital health literacy, especially among physicians, overall acceptance of DHTs in palliative care was limited—particularly among physicians themselves. While 25–35% of participants reported a positive shift in attitudes due to COVID-19, the majority indicated no change. This finding may be less surprising in the palliative care context than in other medical domains: many palliative care patients have limited mobility, severe illness, or high symptom burden, and the need for remote support predates the pandemic. The lack of attitude change therefore does not necessarily indicate resistance to DHTs, but may rather reflect that the perceived need for digital solutions already existed independently of the pandemic.

Professionals identified clear benefits of DHTs, particularly in terms of flexibility and time efficiency. However, structural barriers—such as inadequate infrastructure and limited digital skills among patients and families—remain key obstacles. Data protection and usability concerns were also noted, especially among nurses. Notably, those using conventional digital tools were significantly more likely to adopt innovative ones, suggesting that familiarity may facilitate broader uptake.

These findings underscore the selective and cautious integration of DHTs in palliative care. While professionals recognize their potential, practical and ethical concerns shape adoption. The data highlight the importance of context-sensitive, needs-oriented implementation strategies that address infrastructure, training, and stakeholder acceptance—particularly in underserved rural regions.

### Comparison with prior research

Recent innovative studies have underscored the potential of DHTs across various stages of the palliative care pathway. Notably, the study by Greer and Temel [18] demonstrated how telehealth can effectively facilitate early integration into palliative care. Further evidence from outpatient pediatric palliative care suggests that telehealth can support high-quality palliative care delivery. In a retrospective chart review, Williams et al. found that health outcomes, end-of-life quality metrics, and encounter-level quality indicators were similar across telehealth and in-person care, although access to telehealth varied by language and patient portal use [19]. Similarly, machine-learning approaches show great promise in predicting palliative care needs, raising hopes for earlier and more targeted interventions [20–22]. Innovative technologies are also being explored for patient monitoring. For example, the feasibility of radar technologies for contactless heart rate monitoring in palliative care has been demonstrated, suggesting potential for non-invasive patient monitoring [23]. Other exciting domains include the use of virtual reality to enhance palliative care experiences [24] and the examination of digital legacy management after death [25], which opens new avenues for addressing psychosocial and ethical dimensions in this field.

While such pioneering approaches emphasize the possibilities of DHTs, the review by Finucane et al. [13] revealed that most studies on DHT use in palliative care focus on relatively conventional technologies, such as videoconferencing, electronic health records, and phone-based approaches. Our study deviates from these controlled research contexts by providing real-world insights from daily palliative care practice. Similar to Finucane et al.‘s findings, our results indicate that conventional DHTs, such as telephones and fax machines, are far more commonly utilized than innovative technologies. Our findings are also consistent with earlier qualitative research on users’ perceptions of DHTs in palliative care. These studies revealed a prevailing “low tech, high touch” ethos among palliative care providers [11]. This principle reflects the prioritization of personal and holistic care, which may explain why our survey data showed lower acceptance of DHTs in palliative care compared to general healthcare settings.

It is well-established that hospitals generally have more advanced technical infrastructures compared to outpatient care settings. However, we had anticipated that DHT would play an especially crucial role in outpatient care by facilitating communication among HCPs and bridging geographical distances in patient communication. Contrary to our expectations, inpatient settings reported a wider variety of DHTs in use. It is essential to note, however, that these findings do not account for the frequency of use.

The results on eHealth literacy show different levels within different professional groups in palliative care. These findings largely correspond to the current study situation, with doctors generally having a higher level of digital health literacy than nurses [26]. These differences can possibly be attributed, among other things, to the different training backgrounds, access to digital resources and the different integration of digital health technologies in everyday care.

In line with other studies from other medical domains [27–29], our observed association between urban locations and more frequent use of DHTs underscores the persistent barriers in rural areas, including infrastructural limitations and inadequate mobile data coverage. These obstacles likely hinder the adoption and utilization of DHTs in less densely populated regions.

Several mechanisms may explain the observed differences in DHT use. First, financial and regulatory factors may play a role, as DHT use may not be consistently reimbursed or otherwise incentivised in routine palliative care. Second, organisational models may influence uptake: without structured workflows for digital communication, monitoring, and escalation, HCPs may be reluctant to adopt systems that increase rather than reduce workload. For example, monitoring technologies may require clearly defined responsibilities, triage processes, or dedicated staff to filter and escalate alerts. Third, competencies and institutional support are likely to be relevant, as HCPs may lack not only technical skills but also protected time, training opportunities, and organisational encouragement to acquire them. Our study was not designed to test these hypotheses, but they represent important avenues for future research. Future studies should use mixed-methods designs that combine structured surveys on reimbursement awareness, organisational support, workflow integration, and training opportunities with qualitative interviews to understand motivational, educational, and organisational barriers to DHT adoption.

To effectively integrate innovative DHTs into routine palliative care, systemic barriers such as infrastructure deficits, digital literacy among HCPs and patients, and the perceived lack of relevance of these technologies in sensitive care contexts must be addressed. Moreover, there is a need for a broader discourse within the research and care communities: Which digital approaches are effective in palliative care? And which are ethically acceptable from the perspectives of patients, family caregivers, and HCPs?

Addressing infrastructure, training, and ethical concerns is key to equitable digital integration. Additionally, ensuring the equitable distribution of resources and training is vital to overcoming the digital divide between urban and rural areas, thereby enabling the widespread and effective use of DHTs. Policymakers must prioritize these efforts as digital solutions become increasingly central to healthcare delivery.

### Strengths and limitations

The study has several limitations that should be taken into account when interpreting the results. Firstly, the use of self-completed questionnaires carries the possibility of self-selection bias. In addition, the study was conducted exclusively in one region of Germany, which may limit the generalizability of the results to other regions or countries with different healthcare systems, digital infrastructure or cultural attitudes towards DHTs. The sample may not fully represent the diversity of HCPs in Germany. Another limitation is that the survey did not explicitly assess the specific patient groups cared for by participating HCPs. Consequently, we were unable to stratify attitudes toward and use of DHTs by patient population. This is relevant because palliative care is heterogeneous, and the perceived appropriateness, benefits, and barriers of DHTs may differ depending on the patient groups served. In addition, The use of self-reported data may have introduced recall and social desirability bias. Although the questionnaire was pre-tested to ensure clarity and relevance, there remains a risk that participants may have interpreted the questions differently, which may have impacted the consistency and reliability of the data collected. Another limitation concerns the wording of the survey item assessing the perceived appropriateness of DHTs. The question was posed in general terms without specifying particular types of technologies (e.g., teleconsultations, mobile apps, wearables, documentation tools). As a result, participants may have interpreted the item differently based on their own experiences and associations. This lack of specificity limits the comparability of responses and may have introduced variability that is not attributable solely to professional background or care setting. The survey also did not distinguish whether DHTs were used directly by patients or mediated through relatives or family caregivers. This is relevant in palliative care, where patients may be bedridden, cognitively impaired, or otherwise unable to interact independently with digital technologies. In such situations, family caregivers may be the relevant digital actors. Future studies should therefore differentiate between patient-led and caregiver-mediated DHT use. Finally, while the survey captured perceived barriers to DHT use, it did not allow for an in-depth analysis of why individual HCPs reject or do not use specific DHTs. Reasons for non-use may include lack of perceived relevance, ethical concerns, workflow incompatibility, insufficient infrastructure, reimbursement issues, data protection concerns, or previous negative experiences with digital tools. In addition, an item on practice setting (e.g., single-handed practice, group practice, medical care centre, or hospital) was collected but could not be meaningfully analysed: it applied mainly to ambulatory physicians and thus showed high missingness, and it overlapped conceptually with care structure, which was retained instead.

## Conclusion

This study provides the first quantitative insight into the current use, acceptance, and perceived challenges of DHTs from the perspective of HCPs working in palliative care across a federal state in Germany. The results show that digital practice is still largely based on conventional technologies such as email, fax, and documentation tools, whereas innovative technologies such as video consultations, mobile apps, and wearables remain rarely used. This suggests that digital transformation in palliative care should not primarily start with the introduction of complex new technologies, but with strengthening and systematically expanding existing digital routines.

While healthcare professionals generally express moderate openness to DHTs, acceptance is lower in the specific context of palliative care, and usage appears to be shaped more strongly by structural and demographic factors than by attitudes alone. Therefore, implementation strategies should go beyond efforts to improve acceptance and address the practical conditions of use, including infrastructure, workflow integration, data protection, reimbursement, and technical support. Particular attention is needed for outpatient and rural care settings, where structural barriers may further limit DHT uptake.

These findings suggest that digital transformation in palliative care requires setting-specific and ethically sensitive implementation strategies that account for the realities of serious illness, limited patient mobility, caregiver involvement, and heterogeneous patient needs. Future research should move beyond HCP-reported data and examine patient-led and caregiver-mediated DHT use across different palliative care populations, the reasons for HCP non-use or rejection of specific DHTs, and the actual outcomes of DHT integration in routine care.

## Electronic supplementary material

Below is the link to the electronic supplementary material.

Supplementary material 1

Supplementary material 2

## Data Availability

All data relevant to the study are included in the article or uploaded as supplementary information. For further questions regarding the reuse of the quantitative data, please contact Felix Mühlensiepen (felix.muehlensiepen@mhb-fontane.de).

## Abbreviations

DHT: Digital Health Technology
HCP: Healthcare Professional
SOPC: Specialised Outpatient Palliative Care
eHEALS: eHealth Literacy Scale
PCA: Principal Component Analysis
KMO: Kaiser-Meyer-Olkin Measure
MWU: Mann-Whitney U Test
ANOVA: Analysis of Variance
