# Supplementary material for: Digital health in palliative care: use is largely limited to conventional technologies – a cross-sectional survey of healthcare professionals
Source: BMC Health Serv Res. 2026 Jun 23;26:870. doi: 10.1186/s12913-026-14990-5 (PMC13296108; doi:10.1186/s12913-026-14990-5)
Supplement: Supplementary file 1 — Supplementary material 1 [file 12913_2026_14990_MOESM1_ESM.pdf]

Supplementary Material 1

Conceptual Definitions of Items Addressing the Perceived Potentials and Challenges of Digital Health Technologies

**Potentials**	
**Item**	**Conceptual definition**
Better preparation for patient conversations	Using digital tools to review patient data or symptom reports before consultations, enabling more focused and informed conversations.
More flexibility	The ability to adapt work schedules, communication, or documentation processes more flexibly using digital tools.
Accessibility	Ease of accessing digital technologies or services, regardless of geographic or institutional constraints.
More opportunities to access information, diagnostics and therapy	Improved access to clinical data, diagnostic tools, or therapeutic interventions via digital platforms.
Time savings	Reduction of time spent on administrative or clinical processes due to digital support.
Cost savings	Reduction of financial costs, either directly (e.g., reduced travel) or indirectly (e.g., more efficient workflows).
More accurate documentation	Higher accuracy in documentation through structured digital systems, reducing errors or
Time-independent usage	The possibility to use digital tools outside of traditional working hours (e.g., evenings, weekends).
Location-agnostic usage	The ability to access or use digital technologies regardless of the user's physical location.

**Challenges**	
**Item**	**Conceptual definition**
No need as satisfied with current analog solutions	No perceived need for digital alternatives because current analog processes are considered sufficient.
Lack of knowledge among other caregivers	Insufficient digital knowledge among colleagues or team members, hindering effective collaboration.
Lack of knowledge among relatives	Limited understanding of digital tools among family caregivers, potentially affecting coordination.
Lack of knowledge among patients	Limited understanding of digital tools among patients.
Lack of technical equipment	Absence or inadequacy of required digital infrastructure (e.g., devices, internet, software).

High costs	Perceived or actual financial barriers to obtaining or implementing digital solutions.
Lack of accessibility	Limited ability to access digital tools due to geographical, technical, or institutional constraints.
Lack of user-friendliness	Complex or non-intuitive digital interfaces that reduce usability and adoption.
Gaps in data protection	Concerns about the protection of sensitive data, such as patient records, when using digital systems.
Poor quality of current offers	Perception that currently available digital tools are not of sufficient quality or reliability.
Too little evidence for the benefits of the offers	Lack of scientific evidence supporting the effectiveness or benefits of digital technologies.
Limited information about available offers	Lack of knowledge about which digital technologies are available or applicable in a specific care context.
