# Supplementary material for: Digital health in palliative care: use is largely limited to conventional technologies – a cross-sectional survey of healthcare professionals
Source: BMC Health Serv Res. 2026 Jun 23;26:870. doi: 10.1186/s12913-026-14990-5 (PMC13296108; doi:10.1186/s12913-026-14990-5)
Supplement: Supplementary file 2 — Supplementary material 2 [file 12913_2026_14990_MOESM2_ESM.pdf]

This first part of the survey addresses current usage patterns of information and communication technologies. Please indicate whether you have used the following devices or digital health services in palliative care:

**Multiple choice possible**	Used before COVID-19	Currently using	Will use in future	Not interested	Not currently using	Do not know it
E-Mail	O	O	O	O	O	O
Computer / Notebook	O	O	O	O	O	O
Video consultation	O	O	O	O	O	O
Digital health applications (e.g. prescribable medical apps)	O	O	O	O	O	O
Other mobile health apps	O	O	O	O	O	O
Symptom checkers (e.g. Ada)	O	O	O	O	O	O
Wearables (e.g. smartwatches)	O	O	O	O	O	O
Online pharmacy	O	O	O	O	O	O
PalliDoc (or other documentation software)	O	O	O	O	O	O
Fax machine	O	O	O	O	O	O

Now we will ask you questions about digital health literacy. Please think about your ability to find, understand, and use health-related information from the Internet. Please mark the response that best applies to you.

	Strongly disagree	Disagree	Neutral	Agree	Strongly agree
I know what health resources are available on the Internet.	O	O	O	O	O
I know where to find helpful health resources on the Internet.	O	O	O	O	O
I know how to find helpful health resources on the Internet.	O	O	O	O	O
I know how to use the Internet to answer my questions about health.	O	O	O	O	O
I know how to use the health information I find on the Internet to help me.	O	O	O	O	O
I have the skills I need to evaluate the health resources I find on the Internet.	O	O	O	O	O
I can tell high quality health resources from low quality health resources on the Internet.	O	O	O	O	O
I feel confident in using information from the Internet to make health decisions.	O	O	O	O	O

I consider the use of digital health technologies (e.g., medical apps, video consultations) in healthcare in general to be appropriate.

Not at all	Disagree	Neutral	Agree	Completely agree
O	O	O	O	O

I consider the use of digital health technologies (e.g., medical apps, video consultations) in palliative care to be appropriate.

Not at all	Disagree	Neutral	Agree	Completely agree
O	O	O	O	O

Has your attitude toward digital health technologies changed due to COVID-19?

Yes, it became more positive	Yes, it became more negative	No, it has not changed
O	O	O

Which benefits do you see in using digital health technologies? (Multiple answers possible)
○ Location-agnostic use
○ Time-independent use
○ More accurate documentation
○ Cost savings
○ Time savings
○ More access to information, diagnostics, and therapy
○ Accessibility
○ Greater flexibility
○ Better preparation for patient conversations
○ None
○ Other, please specify: ____________________________

Which barriers do you see in using digital health technologies? (Multiple answers possible)
○ Lack of information about available services
○ Too little evidence for effectiveness
○ Poor quality of current services
○ Data protection concerns
○ Poor usability
○ Lack of accessibility
○ High costs
○ Insufficient technical infrastructure (e.g., weak internet, outdated devices)
○ Lack of knowledge among patients
○ Lack of knowledge among family caregivers
○ Lack of knowledge among colleagues
○ No need, as current analog solutions are satisfactory
○ Other, please specify: ____________________________

This final section includes questions on your background. The data will help us analyze the results of the survey. The survey is anonymous, and no personal identifiers are collected.

**How old are you?**	**How many years have you been working in your profession?**
______ years	______ years

What is your gender?

female	male	diverse

O	O	O

Size of the town/city where you work:

Rural area (under 5.000 inhabitants)	Small town (5.000 – 20.000 inhabitants)	Medium-sized town (20.001 – 100.000 inhabitants)	Large city (über 100.001 – 1 Mio. inhabitants)	Metropolitan city (over 1 Mio. inhabitants)
O	O	O	O	O

Employment status:

Employed	Self-employed	both
O	O	O

Work setting:

Solo medical practice	Group practice	Medical care center	Clinik	Specialized outpatient palliative care
O	O	O	O	O

Your professional role:

- O Hospice nurse
- O Specialized outpatient palliative care nurse
- O Hospital nurse
- O Physician
- O Psycho-oncologist
- O Coordinator
- O Volunteer
- O Social worker
- O Other:__________________

Main care structure in which you work:

- O Specialized outpatient palliative care
- O General outpatient palliative care
- O Specialized inpatient palliative care
- O General inpatient palliative care
- O Inpatient hospice
- O Outpatient hospice service
- O Other:__________________
